# Single-center experience of thoracoscopic sympathectomy for palmar hyperhidrosis with long-term postoperative questionnaire survey

**DOI:** 10.1007/s11748-024-02034-w

**Published:** 2024-04-27

**Authors:** Megumi Kobayashi, Yosuke Kumaya, Yasumiko Hirayama, Hiromi Oda, Hiroyuki Cho, Cheng-long Huang

**Affiliations:** Department of Thoracic Surgery, Tazuke Kofukai Medical Research Institute, Kitano Hospital, 2-4-20 Ohgimachi, Kita-Ku, Osaka, 530-8480 Japan

**Keywords:** Thoracoscopic sympathectomy (TS), Palmar hyperhidrosis, Compensatory hyperhidrosis, Quality of life

## Abstract

**Objectives:**

Thoracoscopic sympathectomy is an effective treatment for palmar hyperhidrosis. However, compensatory hyperhidrosis occurs frequently as a postoperative complication of the procedure. The goal of this study was to elucidate the clinical significance of thoracoscopic sympathectomy using our surgical procedure.

**Methods:**

Consecutive 151 patients who underwent thoracoscopic sympathectomy for palmar hyperhidrosis were studied. In addition, to investigate patients’ satisfaction and long-term quality of life, 111 patients were asked to complete a mailing questionnaire survey, and 84 responded (response rate of 75.7%).

**Results:**

All of the 151 patients reported a reduction in palmar sweating during the immediate postoperative period. None of the patients had pneumothorax, hemothorax, Horner’s syndrome, or worsening of bradycardia. Based on the questionnaire, the surgical success rate was 98.8%. None of the patients had a recurrence of palmar hyperhidrosis during the long-term postoperative period. However, compensatory hyperhidrosis was reported in 82 patients (97.6%). In total, 94.0% of patients had high levels of postoperative satisfaction.

**Conclusions:**

Thoracoscopic sympathectomy is an effective surgical treatment for palmar hyperhidrosis. By contrast, the careful preoperative explanation of compensatory hyperhidrosis is considered to be very important.

## Introduction

Palmar hyperhidrosis is a sympathetic disorder defined by excessive secretion of exocrine glands on the palms [1], and it affects 0.6–10.4% of the general population [2, 3]. It is caused by hyperfunctioning of the sympathetic nervous system and is frequently related to triggering emotional disturbances. The severe symptoms can cause embarrassment as well as social and psychological problems, resulting in a negative impact on patients’ quality of life [4, 5]. Although there are several conservative medical treatments, such as anticholinergic drugs, and botulinum toxin A injections [6, 7], they only have a transient effect.

In contrast, thoracoscopic sympathectomy (TS) has been reported to provide a permanent solution [8, 9]. The success rate of TS in hyperhidrosis has been reported to be over 95% [10–12], and TS involves minimal trauma as well as low morbidity and mortality [7, 13]. Therefore, TS is considered the most effective treatment for hyperhidrosis worldwide [8, 9].

However, compensatory hyperhidrosis occurs frequently as a postoperative complication of TS [12–14]. Because palmar hyperhidrosis is a benign disorder, the clinical purpose of TS is to enhance patients’ quality of life and their satisfaction [15–17]. Taking these findings into consideration, in order to elucidate the clinical significance of our TS procedure for palmar hyperhidrosis, we evaluated the perioperative results as well as postoperative complications among patients who underwent TS for palmar hyperhidrosis in our center. In addition, we investigated patients’ satisfaction and long-term quality of life using a questionnaire.

## Patients and methods

### Patients

Consecutive 151 patients who underwent TS for palmar hyperhidrosis at the Department of Thoracic Surgery, Kitano Hospital, Japan, between January 2007 and December 2022 were studied. Patients included 74 males and 77 females. The age at surgery ranged from 12 to 59 years old (mean 24.0 ± 9.6 years old). This study was approved by the institute’s Ethics Committee (P220700900) and was conducted in accordance with the principles of the Declaration of Helsinki. Written informed consent was obtained from each patient. The patients’ medical records, including perioperative information, were fully documented.

### Surgical technique

**Fig. 1:**
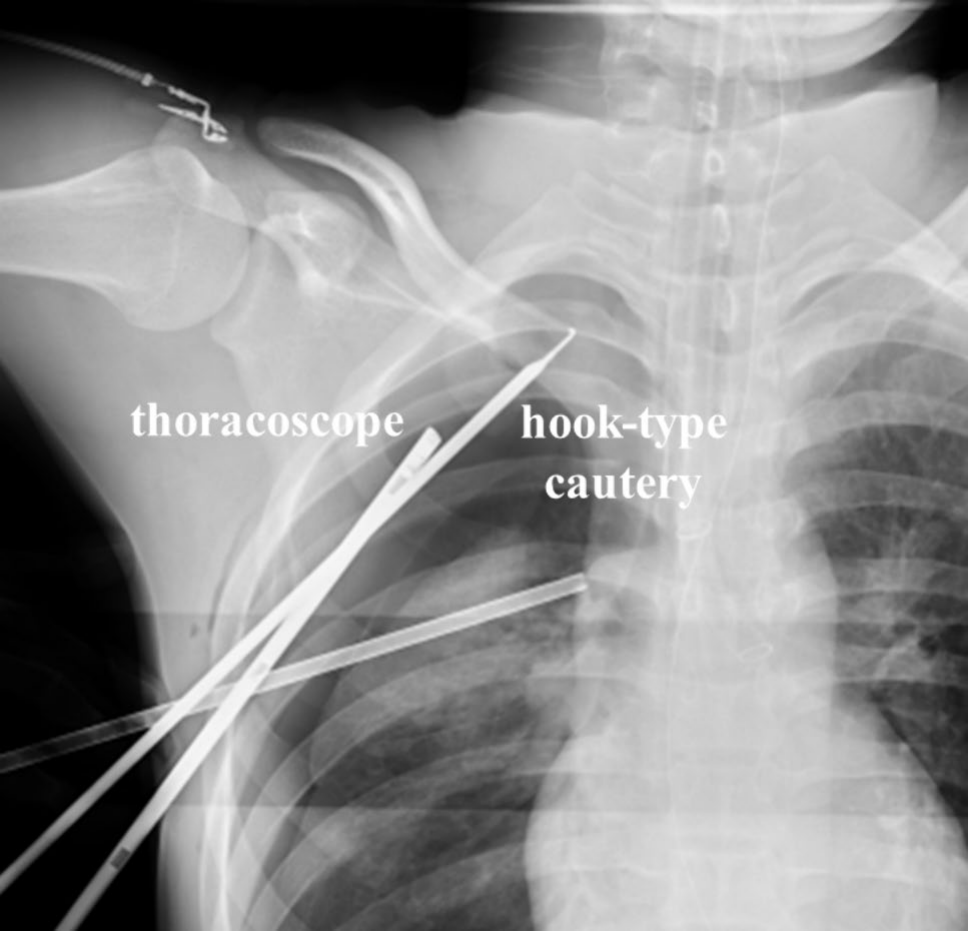
Intraoperative chest roentgenogram confirming the third rib

In general, we performed bilateral TS. However, unilateral TS was performed for patients with preoperative bradycardia or their fear of compensatory hyperhidrosis. Patients were set in a supine position. Following general anesthesia, patients were intubated with a double-lumen tube. A first incision of approximately 1.2 cm in length was made in the fourth intercostal space at the middle axillary line, and the thorax was assessed using a 30° 5-mm thoracoscope. Then, a second incision of approximately 5 mm was made in the third intercostal space at the anterior axillary line for application of an endoscopic hook-type cautery. A 5 mm endoscopic instrument was typically used to compress the lung through the first incision to maintain the field of intraoperative vision (Fig. 1).

After the identification of the sympathetic chain, the location of the third rib was confirmed by chest roentgenogram (Fig. 1). The sympathetic chain was sectioned by cauterizing the bodies of the third and fourth ribs using an endoscopic hook-type cautery. This was followed by thermoablation of the sympathetic chain between the level of the lower border of the third rib and the level of the upper border of the fourth rib. During this step, care was taken not to injure adjacent vessels. To reduce postoperative pain, an intercostal nerve block with levobupivacaine was used. After inserting a chest tube, bilateral ventilation was maintained for 15 min. The same procedure was then performed on the contralateral chain in bilateral surgical cases (129 of 151; 85.4%). After confirming no pneumothorax or hemothorax using the postoperative chest roentgenogram, chest tubes were removed in the operation room. The patient’s vital signs were monitored for 24 h postoperatively.

### Questionnaire survey

**Fig. 2:**
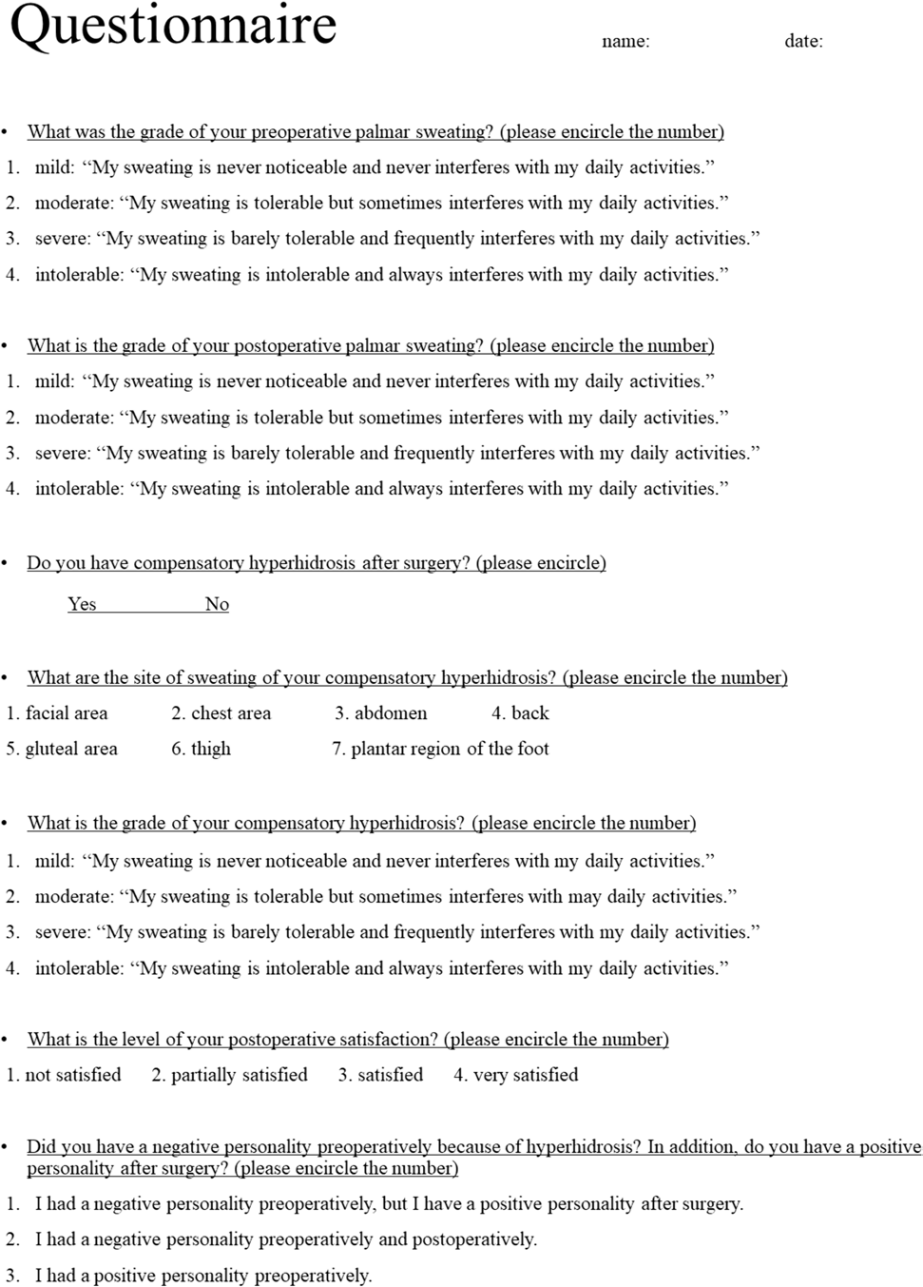
Questionnaire form

In order to investigate postoperative patients’ satisfaction and long-term quality of life, a mailing questionnaire survey was performed on 111 patients who underwent TS for palmar hyperhidrosis at the Department of Thoracic Surgery, Kitano Hospital between January 2017 and July 2022. The patients were asked to provide information on the effect of TS on palmar sweating during the long-term postoperative period, compensatory hyperhidrosis, postoperative satisfaction, and long-term quality of life, including changes in personality (Fig. 2). The first survey was administered in August 2022, and responses were acquired from 53 patients. The second survey was administered to the remaining 58 patients in December 2022, and responses were acquired from 16 patients. The third survey was administered to the remaining 42 patients in April 2023, and responses were acquired from 15 patients. In total, responses were acquired from 84 patients (response rate of 75.7%). The postoperative period for the questionnaire was ranged from 3 to 73 months (mean 29.6 ± 14.7 months).

### Statistical analysis

The degree of sweating was classified according to the hyperhidrosis disease severity scale (grade 1 = mild: “My sweating is never noticeable and never interferes with my daily activities”, grade 2 = moderate: “My sweating is tolerable but sometimes interferes with may daily activities”, grade 3 = severe: “My sweating is barely tolerable and frequently interferes with my daily activities”, and grade 4 = intolerable: “My sweating is intolerable and always interferes with my daily activities”) [18]. The level of postoperative satisfaction was scored between 1 and 4 (level 1: not satisfied, level 2: partially satisfied, level 3: satisfied, and level 4: very satisfied). Categorical variables were compared using *χ*^2^ tests. All *p* values were based on a 2-sided statistical analysis, and a *p *value < 0.05 was considered statistically significant.

## Results

### Perioperative results

Of the151 consecutive patients, 129 (85.4%) underwent bilateral TS, and 22 (14.5%) underwent unilateral TS because of preoperative bradycardia (in 15 patients) or their fear of compensatory hyperhidrosis (in 7 patients). The operation time was 101.6 ± 17.1 min for bilateral TS, and 64.3 ± 21.5 min for unilateral TS. The amount of bleeding was 3.7 ± 4.7 ml, and no thoracotomy was performed for intraoperative hemostasis.

During the immediate postoperative period, all of the patients reported a reduction in palmar sweating. With respect to early postoperative complications, no patient had pneumothorax, hemothorax, Horner’s syndrome, or worsening of bradycardia.

### Effects of TS on palmar sweating during the long-term postoperative period

**Fig. 3:**
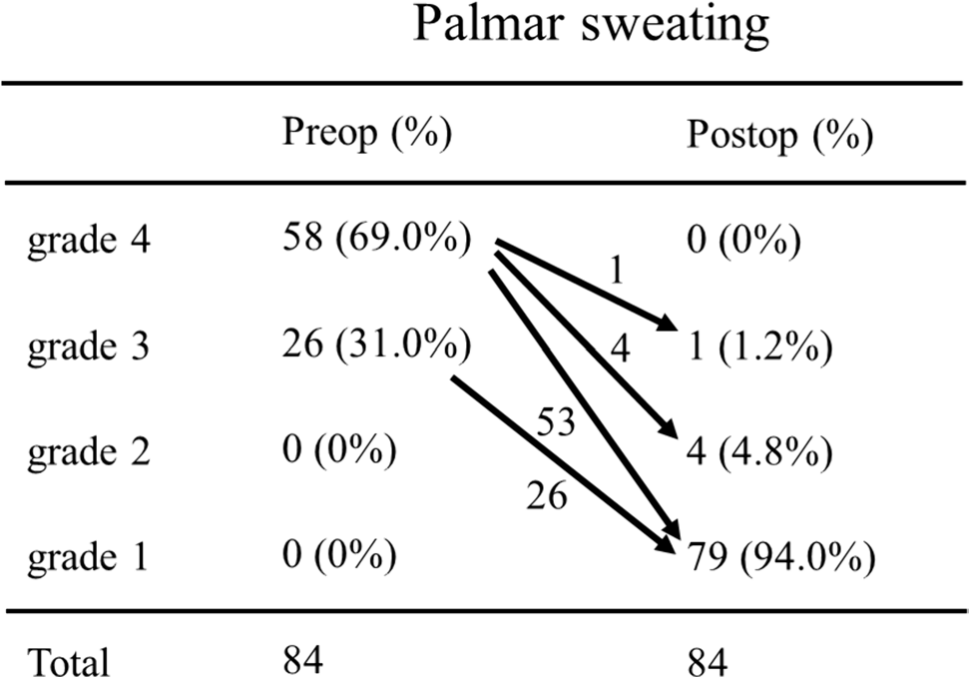
Change in the degree of preoperative palmar sweating and the degree of postoperative palmar sweating. Preop: preoperative; Postop: postoperative; Sweating degree: grade 1 = mild: “My sweating is never noticeable and never interferes with my daily activities”, grade 2 = moderate: “My sweating is tolerable but sometimes interferes with may daily activities”, grade 3 = severe: “My sweating is barely tolerable and frequently interferes with my daily activities”, grade 4 = intolerable: “My sweating is intolerable and always interferes with my daily activities.”

Based on the questionnaire survey of the 84 patients, with respect to the preoperative palmar sweating, 26 of them (31.0%) were at grade 3, and 58 (69.0%) were at grade 4. On the other hand, with respect to postoperative palmar sweating, 79 of the patients (94.0%) were at grade 1, 4 patients (4.8%) were at grade 2, and one patient (1.2%) was at grade 3. The change from the degree of preoperative palmar sweating to the degree of postoperative palmar sweating was grade 4 to grade 1 in 53 patients (63.1%), grade 3 to grade 1 in 26 patients (31.0%), grade 4 to grade 2 in 4 patients (4.8%), and grade 4 to grade 3 in 1 patient (1.2%) (Fig. 3). The surgical success rate (change in the degree of palmar sweating: grade 4 to grade 1, grade 4 to grade 2, and grade 3 to grade 1) was 98.8%. No patient had a recurrence of palmar hyperhidrosis during the long-term postoperative period.

### Compensatory hyperhidrosis

According to the questionnaire survey administered to 84 patients, compensatory hyperhidrosis occurred in 82 patients (97.6%). With respect to the sweating site, compensatory hyperhidrosis of the back was the most common, affecting 68 patients (81.0%). Compensatory hyperhidrosis also occurred in the thigh in 50 patients (59.5%), the abdomen in 47 patients (56.0%), the plantar region of the foot in 33 patients (39.3%), the chest area in 7 patients (8.3%), the facial area in 7 patients (8.3%), and the gluteal area in 4 patients (4.8%). The degree of sweating in compensatory hyperhidrosis was grade 1 in 2 patients (2.4%), grade 2 in 38 patients (45.2%), grade 3 in 34 patients (40.5%), and grade 4 in 10 patients (11.9%) (Table 1). There was no significant association between the sweating degree in compensatory hyperhidrosis and the degree of preoperative palmar sweating (*p* = 0.12) or the degree of postoperative palmar sweating (*p* = 0.40).

**Table 1:** Compensatory hyperhidrosis in relation to preoperative palmar sweating and postoperative palmar sweating among 84 patients

		Compensatory hyperhidrosis				
	n	Grade 1	Grade 2	Grade 3	Grade 4	*p* value
Preoperative palmar sweating						
Grade 3	26	2	14	8	2	0.12
Grade 4	58	0	24	26	8	
Postoperative palmar sweating						
Grade 1	79	2	36	32	9	0.40
Grade 2	4	0	2	2	0	
Grade 3	1	0	0	0	1	
Number of patients						
	84	2	38	34	10	

### Postoperative satisfaction and long-term quality of life

Based on the questionnaire survey of the 84 patients, postoperative satisfaction was at level 4 in 75 patients (89.3%), level 3 in 4 patients (4.8%), level 2 in 3 patients (3.6%), and level 1 in 2 patients (2.4%). As shown in Table 2, the postoperative level of satisfaction was significantly associated with the postoperative degree of palmar sweating (*p* = 0.018) and the degree of sweating in compensatory hyperhidrosis (*p* < 0.001).

**Table 2:** Postoperative satisfaction in relation to postoperative palmar sweating and compensatory hyperhidrosis among 84 patients

		Postoperative Satisfaction				
	n	Level 1	Level 2	Level 3	Level 4	*p* value
Postoperative palmar sweating						
Grade 1	79	1	2	4	72	0.018
Grade 2	4	0	1	0	3	
Grade 3	1	1	0	0	0	
Compensatory hyperhidrosis						
Grade 1	2	0	0	0	2	<0.001
Grade 2	38	0	0	1	37	
Grade 3	34	0	2	0	32	
Grade 4	10	2	1	3	4	
Number of patients						
	84	2	3	4	75	

Additionally, of the 84 patients, 50 patients (59.5%) had a negative personality preoperatively, and, of these 50 patients, 45 (90.0%) reported to have a positive personality after TS.

## Discussion

The severe symptoms of palmar hyperhidrosis can cause embarrassment and social and psychological problems [5]. Although there are conservative medical treatments [6, 7], TS has been reported to provide a permanent solution with a success rate of over 95% [10–12]. Therefore, TS is considered the most effective treatment for palmar hyperhidrosis and is performed worldwide [8, 9].

However, compensatory hyperhidrosis is the most common complication of TS. In fact, compensatory hyperhidrosis was observed in 97.6% of the patients in the present study. Therefore, many studies have discussed the optimal level of sympathectomy for palmar hyperhidrosis, which is an important issue. Yazbek et al. [12] reported that T3 sympathectomy presented compensatory hyperhidrosis with less severity than T2 sympathectomy. In a meta-analysis, Cerfolio et al. [18] also reported that compensatory hyperhidrosis was better after T3 and T4 sympathectomy compared to T2 sympathectomy. Dogru et al. [19] recently reported that patients who underwent T2–T4 sympathectomy had a lower quality of life than patients who underwent T3 or T3–T4 sympathectomy. In addition, Deng et al. [20] found that the surgical success rate was high for sympathectomy performed at the T3 and T3–T4 levels. Therefore, T2 sympathectomy is not considered to be preferable.

However, there are still controversial reports regarding T3 sympathectomy and T4 sympathectomy. Zhang et al. [21] reported that patients’ postoperative quality of life was higher in patients who underwent T4 sympathectomy than in patients who underwent T3 sympathectomy because of reduced complications of compensatory hyperhidrosis. However, Ellatif et al. [22] reported that palmar dryness was less common in patients who underwent T4 sympathectomy than in patients who underwent T3 sympathectomy. In addition, other previous studies have reported that isolated T4 sympathectomy does not ensure an adequate reduction in palmar sweating [18, 23]. Considering these previous reports, we performed T3 sympathectomy for palmar hyperhidrosis.

With respect to our institution’s TS procedure, patients were set in a supine position, which was a simple and safe position for hemi-lateral ventilation and useful for bilateral surgery. In addition, we used a 2-port approach to maintain a good field of intraoperative vision. Then, we resected the sympathetic chain at the T3 level using cauterization with an endoscopic hook-type cautery as completely as possible while taking care not to injure adjacent vessels. Consequently, no patient had postoperative recurrence of palmar hyperhidrosis in our surgical cases. This is important, as a previous study reported that postoperative recurrence of palmar hyperhidrosis decreases patient satisfaction [24]. In addition, to prevent postoperative complications of Horner’s syndrome, an intraoperative chest roentgenogram was routinely performed to confirm the location of the third rib. In fact, most of the operation time comprised waiting for radiological technologists, but we think that their support was essential for this surgery as it allowed us to prevent postoperative complications of Horner’s syndrome. Furthermore, especially for patients with preoperative bradycardia, cauterization of the body of the fourth ribs was carefully performed while paying attention to an electrocardiogram monitor in real time to avoid the worsening of the bradycardia.

Consequently, the surgical success rate of TS for palmar hyperhidrosis was 98.8% in this study. The high rate of surgical success in the present study might be partly due to our surgical procedure of thermoablation which was almost equal to resection of the sympathetic chain in order to avoid postoperative recurrence of palmar hyperhidrosis. No patient had early postoperative complications, including pneumothorax, hemothorax or Horner’s syndrome. As a result, 94.0% of the patients indicated that they were satisfied in the survey administered during the long-term postoperative period. In particular, 90.0% of the patients who had a negative personality preoperatively had a positive personality after TS, which is considered to be a very important effect of TS for patients with palmar hyperhidrosis. From there results, it can be concluded that our TS surgical procedure is safe and clinically useful.

However, compensatory hyperhidrosis also occurred in our cases, and may be more frequent than in previous reports [15, 18, 19]. This result might be also partly due to our surgical procedure of thermoablation which was almost equal to resection of the sympathetic chain, as described above. Therefore, the careful preoperative explanation of compensatory hyperhidrosis is very important for patients with severe palmar hyperhidrosis who are eager to undergo TS. In addition, Dogru et al. [19] reported that postoperative levels of satisfaction with TS were high even when compensatory hyperhidrosis was observed in most patients. Rodriquez et al. [24] also reported that informing patients of possible side effects before TS is essential. Actually, among 44 patients with grade 3 or 4 compensatory hyperhidrosis in the present study, 39 (88.6%) had level 3 or 4 postoperative satisfaction (Table 2).

Generally, we performed bilateral TS. On the other hand, unilateral TS was performed for patients with preoperative bradycardia or fear of compensatory hyperhidrosis. However, there was no difference in the grade of compensatory hyperhidrosis between patients after bilateral TS and unilateral TS in the present study (data not shown).

## Limitations

The main limitation of the present study was the low response rate for the mailed postoperative questionnaire survey. The level of postoperative satisfaction may have been low among the patients who did not complete the questionnaire. Therefore, a further study involving more patients with a higher response rate in the postoperative questionnaire survey is needed in the future.

## Conclusions

TS at the T3 level is a useful surgical treatment for palmar hyperhidrosis. Our surgical procedure did not result in recurrence of palmar hyperhidrosis. However, compensatory hyperhidrosis frequently occurred in this study. Careful preoperative explanation of compensatory hyperhidrosis is important for patients with palmar hyperhidrosis who are eager to undergo TS.

## Data Availability

The data underlying this article will be shared on reasonable request to the corresponding author.
